# P-1768. Single-cell transcriptomic analysis of cutaneous forms of chronic active Epstein–Barr virus disease

**DOI:** 10.1093/ofid/ofaf695.1938

**Published:** 2026-01-11

**Authors:** Yuto Fukuda, Takako Suzuki, Yoshitaka Sato, Ken-ichi Iwata, Yusuke Okuno, Hiroshi Kimura, Yoshinori Ito, Jun-ichi Kawada

**Affiliations:** Department of Pediatrics, Nagoya University Graduate School of Medicine, Nagoya, Aichi, Japan; Nagoya University Graduate School of Medicine, Nagoya, Aichi, Japan; Department of Virology, Nagoya University Graduate School of Medicine, Nagoya, Aichi, Japan; Department of Pediatrics, Nagoya University Graduate School of Medicine, Nagoya, Aichi, Japan; Nagoya University Graduate School of Medicine, Nagoya, Aichi, Japan; Department of Virology, Nagoya University Graduate School of Medicine, Nagoya, Aichi, Japan; Nagoya University Graduate School of Medicine, Nagoya, Aichi, Japan; Fujita Health University School of Medicine, Toyoake, Aichi, Japan

## Abstract

**Background:**

Hydroa vacciniforme-like lymphoproliferative disorder (HV-LPD) and severe mosquito bite allergy (SMBA) are cutaneous forms of chronic active Epstein–Barr virus (EBV) disease (CAEBV) characterized by clonal proliferation of EBV-infected T or natural killer (NK) cells. SMBA and HV-LPD can progress to systemic CAEBV infection; however, their pathogenesis remains unclear. Here, we performed single-cell RNA sequencing (scRNA-seq) analysis of patients with two types of cutaneous CAEBV.

**Methods:**

This study included five patients with HV-LPD, four with SMBA, and two healthy adult controls. Peripheral blood mononuclear cells (PBMCs) were collected from each patient and used for scRNA-seq. In five cases, the EBER gene was enriched to enhance the detection efficiency of EBV-infected cells. ScRNA-seq data were processed and analyzed using Cell Ranger and Seurat. Monocle3 was used for the trajectory analysis.

**Results:**

A total of 79,830 PBMCs were analyzed, with 2,233–13,487 cells per case. EBV gene expression was detected in 5,827 cells (SMBA; 4,343 cells, 12.7%, HV-LPD; 1,484 cells, 3.7%). In the SMBA cases, EBV-infected cells resided at earlier pseudo-time positions than uninfected cells, suggesting a more immature transcriptional state. Additionally, in cases that progressed to systemic CAEBV, EBV-positive cells were positioned earlier in the pseudo-time trajectory than in non-progressed cases.

**Conclusion:**

By combining scRNA-seq analysis with EBV gene detection, EBV-infected cell populations were identified, and host gene expression profiles between infected and uninfected cells were compared at single-cell resolution. The accumulation of EBV-infected cells in transcriptionally immature states suggests a potential link between CAEBV progression.

**Disclosures:**

All Authors: No reported disclosures

